# The Effect of Organoselenium Compounds on Histone Deacetylase Inhibition and Their Potential for Cancer Therapy

**DOI:** 10.3390/ijms222312952

**Published:** 2021-11-30

**Authors:** Theolan Adimulam, Thilona Arumugam, Ashmika Foolchand, Terisha Ghazi, Anil A. Chuturgoon

**Affiliations:** Department of Medical Biochemistry, School of Laboratory Medicine and Medical Science, College of Health Sciences, Howard College Campus, University of KwaZulu-Natal, Durban 4041, South Africa; theoadimulam@gmail.com (T.A.); cyborglona@gmail.com (T.A.); ashmikafoolchand@yahoo.com (A.F.); terishaghazi@gmail.com (T.G.)

**Keywords:** cancer, organoselenium compounds, selenomethionine, selenocysteine, methylselenocysteine, histone deacetylation

## Abstract

Genetic and epigenetic changes alter gene expression, contributing to cancer. Epigenetic changes in cancer arise from alterations in DNA and histone modifications that lead to tumour suppressor gene silencing and the activation of oncogenes. The acetylation status of histones and non-histone proteins are determined by the histone deacetylases and histone acetyltransferases that control gene transcription. Organoselenium compounds have become promising contenders in cancer therapeutics. Apart from their anti-oxidative effects, several natural and synthetic organoselenium compounds and metabolites act as histone deacetylase inhibitors, which influence the acetylation status of histones and non-histone proteins, altering gene transcription. This review aims to summarise the effect of natural and synthetic organoselenium compounds on histone and non-histone protein acetylation/deacetylation in cancer therapy.

## 1. Introduction

Cancer is a leading cause of mortality worldwide. In 2018, the World Health Organization’s Annual Global Cancer Statistics indicated that 18.1 million new cases and 9.6 million deaths have occurred [1,2]. These statistics have increased tremendously over the past few years and are expected to double by 2040 [2]. The increase in cancer incidence and mortality is due to several factors, such as population growth, aging, and changes in the prevalence and distribution of cancer risk factors, the majority of which are associated with socioeconomic development [1]. To date, significant advances have been made in cancer prevention and therapy; however, early detection, toxic side effects, drug resistance, and treatment costs pose substantial challenges [2].

Lifestyle and dietary modification are key in preventing cancer [2]. Selenium (Se) is a trace element and essential micronutrient that can be obtained through the diet and nutritional supplements. Selenium plays a critical role in cellular physiological processes and is required for the proper functioning of all organisms [3]. In biological systems, selenium acts as a cofactor for enzymatic reactions and is incorporated into amino enzymes and selenoproteins [3]. Selenium also modulates cell survival and proliferation through its pro- and anti-oxidant effects [4,5] and anti-inflammatory effects [6,7,8,9].

Cancer arises from a continuous oxidative and inflammatory environment, and a selenium deficiency has been correlated with increased cancer incidence and mortality [10,11,12]. In contrast, selenium supplementation has been shown to reduce cancer incidence and mortality [13]. There is no doubt that Se compounds can be both advantageous and disadvantageous in cancer. However, this is dependent on minor structural changes in Se to produce favourable analogues. For this reason, natural and synthetic organoselenium compounds and nano-selenium particles have attracted growing interest as potential anti-cancer agents. Organoselenium compounds are usually favoured over inorganic selenium compounds due to their increased bioavailability and decreased toxicity [14]. Similarly, nano-selenium particles have shown greater bioavailability and even lower toxicity than organoselenium compounds [15]. Apart from their decreased toxicity, organoselenium compounds and nano-selenium particles have both shown specificity for cancer cells. This was conferred by their selective uptake, localisation, and accumulation in cancer cells [16]. The selective uptake of Se by cancer cells has been explained in vitro. Gandin et al. explained that the aberrant metabolism of cancer cells may be attributed to the selective uptake of Se. The authors further described that the reduction of selenide to selenite stimulated the uptake of Se by mediating a membrane-associated ATP-dependent transporter [16].

Emerging evidence suggests that the chemopreventative and therapeutic activity of organoselenium compounds and nano-selenium particles may be attributed to alterations in the epigenome [17,18]. These alterations can occur via epigenetic changes, such as histone acetylation. Previous studies have indicated that organoselenium compounds and nano-selenium particles can act as histone deacetylase inhibitors, which alter the acetylation of histones and non-histone proteins, thus regulating gene expression and protein activity [17,18,19]. In this review, we summarise the effect of organoselenium compounds and nano-selenium particles on histone and non-histone protein acetylation/deacetylation in cancer prevention and therapy.

## 2. Cancer and Histone Acetylation

Cancer refers to the uncontrolled growth of abnormal cells that leads to the formation of tumours that can spread to various parts of the body. Cancer occurs from aberrations in gene expression and protein function and is often the consequence of an accumulation in genetic and epigenetic events [20]. Epigenetics refers to the regulation of gene expression by heritable modifications that are independent of the DNA sequence. These modifications include DNA methylation, histone post-translational modifications, and microRNAs. Although not as widely studied as DNA methylation, histone acetylation plays a critical role in cancer development, and, hence, it will be the focus of this review.

In eukaryotes, DNA interacts with histones to form nucleosomes. Each nucleosome comprises an octamer of positively charged histones—2 copies each of H2A, H2B, H3, and H4—around which approximately 147 base pairs of negatively charged DNA are wound [21]. H1 does form part of the histone octamer; however, it serves a crucial role in organising the nucleosomes into higher-order chromatin structures [21]. The structure of chromatin is important in determining gene expression and can be divided into transcriptionally silent heterochromatin or transcriptionally active euchromatin [21,22].

**Figure 1:**
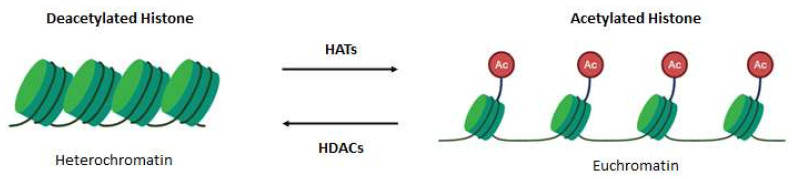
The process of histone acetylation and deacetylation. Histone acetylation is catalysed by histone acetyltransferases (HATs) and is associated with a transcriptionally active chromatin structure (euchromatin). In contrast, histone deacetylation is mediated by histone deacetylases (HDACs) and is associated with a transcriptionally repressed chromatin structure (heterochromatin).

The modification of the amino-terminal ends of histone tails by acetylation or deacetylation influences the interaction between the DNA and histone proteins and thus influences the chromatin structure. Histone acetylation is associated with euchromatin and is catalysed by histone acetyltransferases (HATs), which transfer the acetyl group from acetyl coenzyme A to lysine residues [21,22]. In contrast, histone deacetylases (HDACs) remove acetyl groups, and histone deacetylation is associated with heterochromatin [22] (Figure 1).

Both HATs and HDACs are also able to modify a large variety of non-histone proteins whose activity depends on their acetylation statuses, such as transcription factors, chaperone proteins, signal transduction mediators, structural proteins, and inflammatory mediators [23,24,25]. Consequently, changes in the acetylation status affect protein stability, protein–protein interactions, and protein–DNA interactions [22].

Numerous studies have indicated a role for histone acetylation and deacetylation in cancer [26,27,28,29,30,31]. Clinicopathological analyses of primary non-small cell lung cancer tissues revealed a positive association between lower levels of H3K9ac, H3K18ac, and H4K16ac and tumour recurrence [26,27]. In prostate cancer tissues, the levels of H3ac and H4ac were found to be significantly decreased compared to those in non-malignant prostate tissues [28]. In another cohort study, elevated levels of H3K18ac were correlated with an increased risk of prostate tumour recurrence and relapse [29]. Low levels of H3K18ac, H4K12ac, and H4K16ac were determined to be an early sign of breast cancer. In contrast, low levels of H3K18ac were correlated with a better prognosis of oesophageal squamous cell carcinoma [31]. Moreover, the tumour suppressor and oncogenic activity of various proteins are also dependent on the recruitment of HATs and HDACs, and, thus, acetylation and deacetylation play a vital role in cancer initiation and progression [32,33,34].

## 3. Naturally Occurring Organoselenium Compounds

**Figure 2:**
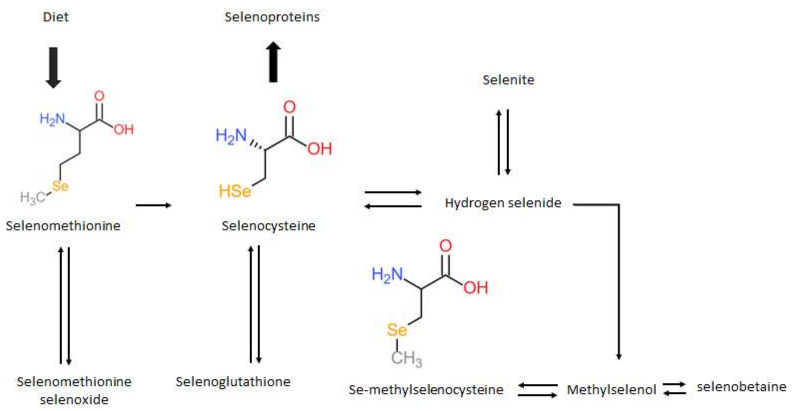
Summary of the metabolism and structures of major dietary organoselenium compounds.

Numerous tools have emerged to facilitate the screening of molecular targets and therapeutic candidates for the identification of compounds associated with histone deacetylation inhibition. These compounds include short-chain fatty acids, hydroxamic acids, benzamides [35,36,37], and other chemical families, such as organoselenium compounds [18]. Selenium is an essential trace element found in the soil, which is absorbed from the diet in two significant forms [38]. Cereal grains and enriched yeast supply selenomethionine (SeMet), while some plants, such as garlic and broccoli, bio-accumulate Se-methyl selenocysteine (MSC) [39]. SeMet is an amino acid containing a sulfur to Se modification most commonly found in nuts, potatoes, and meat proteins, such as fish and chicken [40]. In humans, SeMet is incorporated into proteins by substituting methionine via the acylation of Met-tRNA or the conversion to selenocysteine (SeCys) through a transsulfuration mechanism [41,42]. SeCys can then be cleaved by the enzyme *β*-lyase to form hydrogen selenide (H_2_Se) (Figure 2). SeMet has demonstrated cytotoxicity in lung, colorectal, breast, prostate, and melanoma cancer cells [43,44], highlighting an inverse relationship between Se intake and cancer incidence [38,39]. Although these cytotoxic effects have been observed at a medium to high micromolar range, a strong selectivity towards cancer cells over normal cells has been identified in vitro [45].

The monomethylated seleno–amino acid derivative, more commonly known as MSC, cannot be incorporated into proteins. Instead, it is converted to methylselenol by selenocysteine Se-conjugated *β*-lyases [41,46]. The metabolism of MSC into methylselenol has not yet been identified in animal models or cells owing to the high volatility and reactivity of methylselenol [16]. The cytotoxicity of MSC in vitro has been shown in the micromolar range for human colon, breast, lung, and oral squamous cell lines [43,47], while in vitro treatment with MSC has shown reduced vascular endothelial growth factor expression [43].

### Natural Selenium Compounds and Their α-Keto Acid Metabolites

HDAC inhibitors have demonstrated potential as cancer therapeutic agents since they potentially de-repress epigenetically silenced genes by altering the histone acetylation status [48].

In one study [49], the authors found that methylselenic acid (MSA) increased the acetylation of histone 3 and *α*-tubulin in a time- and concentration-dependent manner. The same group investigated the effect of MSA in a cell-free assay system and cell lines. MSA did not exhibit HDAC inhibitory activity in a cell-free system based on the Flourde Lys™ substrate deacetylation. However, in human non-Hodgkin’s B-cell (DoHH2, DHL4, RL, and SUD4) cell lines, MSA had a concentration-dependent inhibitory effect on HDAC activity [49]. In oesophageal squamous cell carcinoma (ESCC) cells, MSA reduced HDAC activity and up-regulated GCN5 protein levels, which is a transcription-related histone acetyltransferase associated with histone acetylation and gene activation [50].

HDAC inhibition has been reported by MSC and SeMet, which are transaminase substrates of glutamine transaminase K (GTK) and l-amino acid oxidase [51]. However, it was reported that SeMet is a poor substrate for aminotransferase activity as compared to MSC [52,53].

**Figure 3:**
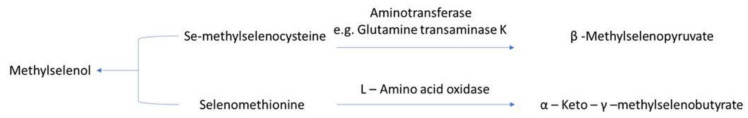
Summary of the formation of α-keto acids.

Previously, it has also been shown that the *α*-keto acid metabolites of organoselenium compounds alter histone deacetylase activity and histone acetylation status [18]. For the *α*-keto acid metabolites to be formed, methylselenol must be created in situ from organoselenium compounds by the action of *β*-lyases, but a transamination reaction must compete with the *β*-elimination for an *α*-keto acid to be formed [54]. In a cell-free system, MSC forms *β*-methylselenopyruvate (MSP) via the enzyme glutamine transaminase K, while SeMet forms *α*—Keto—*γ*–methylselenobutyrate (KMSB) via the enzyme l-amino acid oxidase, as summarised below (Figure 3).

Structurally, MSP and KMSB resemble short-chain fatty acids, a significant class of HDAC inhibitors [53]. These *α*-keto acid metabolites share substantial similarity to butyric acid, which suggests their selectivity for histone deacetylases. Most HDACs hold a coordinating zinc atom in the active site. At the same time, seleno *α*-keto acids possess a highly electronegative selenium moiety in the vicinity of the zinc atom active site, enabling the disruption of the charge relay system within the HDAC pocket [53].

MSP and KMSB exhibit a dose-dependent inhibitory activity on human HDAC1 and HDAC8 in human colon cancer cells [17]. In addition, prostate cancer cells treated with both MSP and KMSB had accumulated acetylated histone H3 [18]. Colon cancer cells treated with MSP and KMSB showed an increase in p21 mRNA and protein expression, and increased histone acetylation associated with the P21WAF1 promotor region [17]. MSC, MSP, and KMSB were able to induce global histone acetylation in prostate, breast, lung, and leukaemia cells, while SeMet did not affect the histone acetylation [52].

## 4. Synthetic Organoselenium Compounds

The synthesis of organoselenium compounds was first reported by Lowig as early as 1836; however, the malodorous nature, troublesome purification, and the instability of many Se derivatives hampered early developments [55]. Research into organoselenium compounds picked up in the 1970s as they were found to be less toxic than their inorganic counterparts and were found to have several useful applications [55,56,57]. Presently, the synthesis and applications of organoselenium compounds are still the centre of intense research and may play a central role in cancer therapeutics [16]. Below, we discuss the role of synthetic organoselenium (methylseleninic acid, seven derivatives of suberoylanilide hydroxamic acid and ebselen) on their HDAC inhibitory and anti-cancer properties.

### 4.1. Methylseleninic Acid

The oxoacid methylseleninic acid (MSA, CH_3_SeO_2_H) is considered among the simplest Se-containing compounds with chemopreventative and chemotherapeutic properties. Due to its pro-oxidant nature, MSA was shown to be effective against human pancreatic [58], lung [59], breast [60], and prostate [61,62] tumour cellular models. MSA has also shown to be effective against rodent mammary [63] and pancreatic [58] in vivo cancer models, as well as colon [64] and prostate cancer [54,58] xenograft models.

**Figure 4:**
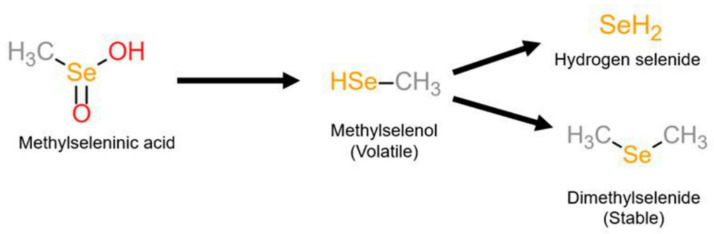
Metabolism of methylselenic acid (MSA). MSA is reduced to the volatile metabolite methylselenol, which is further reduced to hydrogen selenide or methylated to the stable dimethyl selenide.

Contrary to selenoamino acids, MSA circumvents the need for *β*-lyase to generate methylselenol. MSA is easily reduced to methylselenol via enzymatic and nonenzymatic processes [65]. In a reaction with three molecules of thiol, MSA forms selenylsulfide, which is further reduced to methylselenol in the presence of excess thiols [66]. In cells, where glutathione is the major thiol, a methyl-selenium-glutathione intermediate is formed, which undergoes reduction by glutathione reductase to form the key intermediate methylselenol. Methylselenol can undergo demethylation to replete selenoenzymes, producing hydrogen selenide [65], or be further methylated to dimethyl selenide (Figure 4) [67]. The reduction to methylselenol generates superoxide, resulting in cellular dysfunction and death [16]. The redox modifications induced by MSA may contribute to its anti-proliferative and pro-apoptotic effects in cancer cells via caspase activation, ER stress, induction of unfolded protein response, cytochrome c, and PARP cleavage [61,62].

In addition to the pro-oxidative properties of MSA, the inhibition of HDAC activity could be a contributing factor to MSA’s anti-carcinogenic effects. Kassam, Goenaga-Infante [67] were the first to demonstrate the HDAC inhibitory action of MSA in four diffuse large B cell lymphoma cell lines (diffuse large B-cell lymphoma (DLBCL): DoHH2, RL, SUD4, and DHL4). MSA (30 µM, 2 hr) was shown to inhibit both class I and II HDACs by 40–50% as a concentration-dependent increase in the acetylation of H3 (regulated by class I HDACs) and *α* -tubulin (regulated by HDAC6, a class II HDAC) occurred. HDAC activity was also measured using cell-based and cell-free assays. While the activity assays involving intact cells confirmed the concentration-dependent HDAC inhibitory action of MSA in all four cell lines, MSA did not affect HDAC activity in the cell-free assay, which used HeLa nuclear extracts. The authors further demonstrated that medium from cells exposed to MSA had a slight (21%) inhibitory effect on the HDAC activity of HeLa nuclear extracts; however, medium incubated with MSA in the absence of cells had no effect on the activity of HDACs [67]. The above data suggest that the inhibitory action of MSA is likely due to the intracellular activation of MSA to methylselenol, which is responsible for its anti-tumour activity [65]. The volatile nature of methylselenol would explain the small effect observed as it is not retained in the cell medium. Kossam, Goenaga-Infante [67] found that the intracellular Se metabolite formed after MSA exposure to the cell was dimethyl selenide. Although methylselenol was not detectable due to its high volatility, the presence of dimethyl-selenide confirmed that methylselenol was the major metabolite formed and was thus likely responsible for HDAC inhibition.

The authors further hypothesised that inhibition of HDAC activity might be responsible for HIF-1 expression and activity, providing a potential mechanism by which MSA inhibits angiogenesis. However, they did not demonstrate a direct relationship between HDAC activity and HIF-1 expression or activity. It was suggested that the concentration inhibiting HDAC activity was similar to that required for the inhibition of HIF-1*α* expression and VEGF secretion. Thus, HDAC inhibition may be a potential mechanism by which MSA inhibits angiogenesis in vivo, although this claim requires further investigation [67].

The modulation of HDAC activity was further investigated in human oesophageal squamous cell carcinoma cell lines (EC9760 and KYSE-150) exposed to MSA (5 µM; 24 hr) [53]. MSA treatment significantly increased H3 acetylation at lysine 9 (H3K9) and lysine 18 (H3K18); however, no detectable changes were observed at other sites on H3, and the total H3 was only slightly upregulated. H3 hyperacetylation post-MSA treatment was due to the reduced expression of HDAC 1 and 2, impaired HDAC activity, and increased expression of the HAT, general control non-repressed protein 5 (GCN5) [53]. Krüppel-like factor 4 (KLF4) participates in the transcription of various oncogenes and tumour suppressor genes and could either promote or inhibit cell growth in a tissue-dependent manner [68,69]. Overexpression of KLF4 was shown to inhibit growth and invasion of several tumour cell lines [70]; however, it is widely reported to be downregulated in ESCC. MSA treatment increased KLF4 expression via the increased acetylation of H3 at KLF4 promoters in KYSE-150 cells, contributing to MSA-mediated ESCC cell growth inhibition [71].

While the earlier studies examined specific acetylation marks, a recent study by Khalkar, Ali [72] in human chronic myeloid leukaemia K562 cells evaluated and compared genome-wide epigenetic alterations induced by MSA (5 µM, 24 hr) with those of the inorganic Se, selenite (6 µM, 24 hr). Both compounds reduced the global nuclear HDAC activity by 10%; however, these results were not significant. Western blot analysis revealed a significant increase in global H3K9ac upon MSA treatment; these results were not supported in MCF-7 breast cancer cells, which showed that MSA had no effect on H3K9ac [73]. Both studies did observe a negligible effect on H3K9ac by selenite. A chromatin immunoprecipitation assay followed by a whole genome-wide sequencing using the H3K9ac histone mark revealed that the cytotoxic effects exerted by MSA were not solely dependent on its pro-oxidant nature. MSA affected genes related to cell adhesion, glucocorticoid receptor binding, and inositol-3-phosphate synthase activity [72].

The mechanism by which MSA inhibits HDAC activity needs further investigation. Classical HDAC I and II inhibitors contain a side chain that can easily reach the catalytic pocket of HDACs to chelate Zn^2+^ ions found at the active site. Neither MSA nor its metabolites include these features [74]. However, MSA was shown to inhibit other enzymes, such as PKC, via redox modifications to key cysteine residues [75]. There is, therefore, the potential for Se compounds to directly alter the HDAC structure and catalytic activity; however, such a relationship needs further investigation.

### 4.2. Selenoderivatives of Suberoylanilide Hydroxamic Acid 

**Figure 5:**
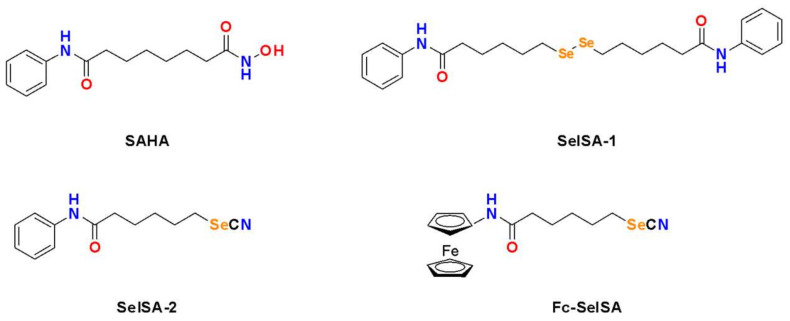
Structures of the HDAC inhibitor SAHA and its selenium-containing derivatives.

Suberoylanilide hydroxamic acid (SAHA, C_14_H_20_N_2_O_3_) or vorinostat is a well-known HDAC inhibitor approved by the USA Food and Drug Administration for treatment against advanced cutaneous T cell lymphoma [76,77]. It has been shown to be effective against other hematological malignancies and is known to block in vitro and in vivo proliferation of cancer cells with little to no toxicity to normal cells [78,79,80,81,82]. The anti-proliferative effect of SAHA is believed to be due to its ability to inhibit HDAC activity, leading to the accumulation of acetylated proteins and histones, thus altering the transcription and activity of multiple genes related to cell cycle arrest, apoptosis, and differentiation [83,84,85]. While SAHA is effective against hematological malignancies, it has limited efficacy in the treatment of solid tumours [86,87]. Se-containing SAHA derivatives have been developed to overcome the shortfalls of SAHA. The most well-investigated SAHA Se-containing analogue includes the Se-dimer SelSA-1, also known as Bis(5-phenylcarbamoylpentyl) diselenide [B(PCP)^−2^Se], and the selenocyanide SelSA-2, also known as 5-phenylcarbamoylpentyl selenocyanide (PCP-SeCN), and a ferrocenyl modified SelSA analogue known as Fe-SelSA (Figure 5).

SelSa-1 and SelSa-2 were developed in 2010 by Desai and co-workers. Its inhibitory activity was evaluated in Hela nuclear extracts and its effectiveness compared against SAHA. Both SelSA-1 (50 nM) and SelSA-2 (50 nM) were shown to be the superior HDAC inhibitors, disrupting HDAC activity by 81% and 95%, respectively, whereas SAHA (500 nM) only inhibited HDAC activity by 77% [88]. Similar results were observed by Gowda Madhunapantula [89] in Hela nuclear extracts. SelSA-1 or SelSA-2 dose-dependently decreased HDAC activity in the WM35 melanocytic lesion cell line, which resulted in the acetylation of histones H3 and H4. SAHA was 50–60% less effective against obstructing HDAC activity compared to SelSA compounds. Moreover, the topical application of the SAHA Se-derivatives was found to kill melanocytic lesions developed on laboratory-generated skin reconstructs two to four times more effectively than SAHA and decreased tumour development by 87% [89]. SelSA-1 and SelSA-2 were also shown to be more effective against lung cancer cell lines (A549, H2126, H1299, H226, H460, H522, H23, and H441) as they exhibited a lower IC_50_ than SAHA and more potent inhibition of growth activity was observed using the Se derivatives. However, normal lung epithelial cells showed resistance to the SelSA-1 and SelSA-2, suggesting that these SelSA compounds will be well tolerated as compared to SAHA. While the effect of these SAHA derivatives against HDAC activity was not directly investigated, the authors believe that the anti-proliferative effects are due to the induction of autophagy and inhibition of MAPK and PI3K signalling, which are common occurrences during HDAC inhibition [90].

The mechanism of SAHA’s inhibitory action on class I and II HDACs is through the chelation of zinc (Zn^2+^) ions present in the active sites of HDACS [91]. Replacing the zinc-binding group (carbonyl and hydroxyl amine group) of SAHA with Se improves its affinity for Zn^2+^ ions, consequently enhancing its effectiveness as an HDAC inhibitor [92]. In silico, modifications with organoselenium to the zinc-binding group of SAHA resulted in 1726 ligands. Further molecular docking simulations revealed that the five best ligands (CC27, HA27, HB28, IB25, and KA7) had better binding affinity and interactions with Zn^2+^ ions in inhibited HDACS than SAHA [93]. In silico molecular docking revealed that SelSA-1 shares the same common binding sites on class I HDACs (class I) with SAHA. However, differential binding patterns of Sel-SA-1 with HDAC2 and HDAC8 were observed. For instance, HDAC2 appears to bind similar to SAHA, where the SeH of SelSA-1 binds deeply to HDAC8. For HDAC8, SelSA-1 mimics the binding of trichostatin, which is another potent HDAC inhibitor against different cancers [94].

Docking simulations further established that SelSA-2 selectivity inhibited HDAC6 as SelSA-2 adopted a favourable binding position in the active site of HDAC6 with the selenocyanide group engaging in key hydrogen bonds critical for chelation of Zn^2+^ ions in the catalytic domain [95]. Hydroxamic acid is able to chelate the Zn^2+^ ion, which can inhibit HDAC activity [96]. This was confirmed in the breast cancer cell lines MCF-7 and MDA-MB-231 as SelSA-2 selectively inhibited HDAC6, resulting in tubulin acetylation. Moreover, SelSA-2 specifically targeted breast tumours in vivo and improved treatment efficacy with fewer side effects compared to SAHA [95]. Modifications to the cap-linker of SelSA-2 with ferrocenyl (FC-SelSa-2) have also demonstrated effectiveness against MDA-MB-231 cells. Molecular docking analysis showed Fc-SelSA formed new hydrogen-bonding interactions with residues D98 and G151, whereas SAHA and SelSA were unable to do so. Moreover, Fc-SelSA was selectively more potent against MDA-MB-231 cells in comparison to MCF-7 cells, with no toxicity against normal cells. In addition, Fc-SelSA showed a relatively low acute toxicity in vivo and significantly inhibited the growth of triple-negative breast cancer in a xenograft mouse model [97]. Given its high HDAC binding affinity and potent therapeutic effect, selenoderivatives of SAHA serve as a highly promising candidate for targeted cancer therapy with clinical translation potential.

### 4.3. Ebselen

Ebselen (C_13_H_9_NOSe), first synthesised in 1924, was considered pharmacologically irrelevant until its capability as a potent anti-oxidant was established in 1984 [98,99,100]. Ebselen mimics glutathione peroxidase to detoxify ROS. ROS oxidises the resting state selenol (Ebselon–SeH) to selenenic acid (Ebselon–SeOH), which is subsequently reduced to active selenol by glutathione via a selenenyl sulphide intermediate (ebselen–SeSG) [101]. The anti-oxidant actions of ebselen are also demonstrated through its ability to react with the thioredoxin system, responsible for removing ROS and reactive nitrogen species (RNS) [102]. Ebselen’s antioxidative properties have been widely studied, suggesting that it might possess anti-proliferative and anti-cancer properties through ROS production [103]. These anti-cancer characteristics may also be regulated by the inhibition of quiescin sulfhydryl oxidase 1 (QSO1), an enzyme that enhances growth and tumour cell invasion and alters the composition of the intracellular matrix [104].

**Figure 6:**
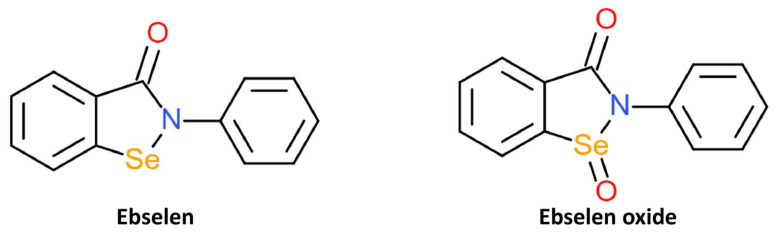
Structures of HDAC inhibitors ebselen and its oxidised derivative, ebselen oxide.

To identify potential and novel HDAC inhibitors, two separate studies screened drug and compound libraries approved by the Library of Pharmacologically Active Compounds (LOPAC), US FDA, and National Institutes of Health Clinical Collection compound library [105,106]. In the first study, 1280 compounds were evaluated for potential inhibitory activity against class I and IIa HDACs. Ebselen was identified as one of five compounds with inhibitory action against class I and Iia HDACs and was most effective against HDAC2 [105]. The screening of 1360 compounds from FDA and National Institutes of Health Clinical Collection library against HDACs from subtypes 1 to 11 also found ebselen to exhibit selective HDAC inhibition [106]. Ebselen was shown to selectively inhibit the activity of HDACs 5, 6, 8, and 9 by more than 50%. The HDAC inhibitory action of eleven ebselen analogues was also investigated; in this review, we focused on the Se-containing ebselen analogue, ebselen oxide (Figure 6). Ebselen oxide was also shown to dose-dependently inhibit HDAC 1, 3, 4, 5, 6, 7, 8, and 9 and increased the potency of HDAC8 inhibition in comparison to ebselen. Unlike other synthetic organoselenium, ebselen and ebselen oxide were shown to effectively inhibit nicotinamide adenine dinucleotide (NAD^+^)-dependent class III HDACs. Ebselen and ebselen oxide dose-dependently inhibited SIRT1, SIRT2, SIRT3, and SIRT5 activities in biochemical assays. The IC50 values of these three compounds on SIRTs were in the range of 0.3 to 6 μM.

Like MSA, ebselen lacks the characteristic features of HDAC inhibitors to chelate Zn^2+^ ions present in the active site of HDACs. Its inhibitory action may also be covalent modification to cysteine residues of HDACs, similar to its irreversible inhibitory action against inositol–monophosphatase (IMPase). The therapeutic potential of ebselen is also explored in infectious diseases, such as SARS-CoV-2. A recent study has shown that ebselen and its derivatives inhibit the main protease of SARS-CoV-2 via ebselen interaction with cysteine [107].

## 5. Selenium Nanoparticles

Over the past three decades, the emergence of nanotechnology has transformed the perception of drug delivery and development by providing many disease pathophysiology and treatment options [108]. Nanotechnology involves sub-microscopic particles or nanoparticles (NPs) with remarkably unique features, such as small size, high surface area, surface charge, surface chemistry, solubility, and multi-functionality [109]. The incorporation of nanoparticles into nutrition is advantageous to solubility, protection from oxidation and enzymatic degradation, extended residence time, and enhanced bioavailability [110].

Biogenic selenium nanoparticles (SeNPs) are biocompatible and less toxic compared to selenate and selenite [111]. However, their toxicity varies among different species [112]. Biogenic SeNPs, with an LD_50_ of 198.1 mg/kg, were reported to be 26-fold less toxic than SeO_2_, with an LD_50_ of 7.3 mg/kg [113]. The use of SeNPs drastically decreased death incurred by Se-associated acute toxicity up to four times in a rodent model [114]. In mice, sub-acute and short-term toxicity studies revealed the higher toxicity of selenite compared to SeNPs. Liver injuries due to a high Se dosage are substantially reduced by SeNPs, as indicated by the hepatotoxicity biomarkers [115].

Due to their high bioavailability and low toxicity, SeNPs are advantageous over their organic and inorganic variants and play a role in biomedical applications, including as anti-oxidants, chemopreventative agents, and anti-cancer drug delivery carriers [116]. By exploiting the overexpression of folate receptors in most cancers, folate has been widely used as a ligand for nanoparticles. Recently, it has been demonstrated that selenium–chitosan–folic acid nanocomplexes selectively bind to the HeLa cell surface, thus mediating gene silencing in vitro [117]. Furthermore, these SeNPs demonstrated low cytotoxicity in non-cancer cell lines in vitro.

The concept of nanomedicine has emerged in therapeutics because it offers unique advantages, such as its enhanced safety [109]. SeNPs have a range of medical applications, including as anti-microbial, anti-oxidant, and anti-cancer agents [118,119]. SeNPs scavenge ROS in a size-dependent manner, where smaller SeNPs hold greater free radical scavenging potential [120]. SeNPs are one of the successfully tried nanoparticles to induce cytotoxicity in cancer cells. SeNPs-based approaches provide hope in fighting drug resistance, mitigating toxicities in chemotherapeutic agents, and transporting chemotherapeutics to their target site [109]. Although the mechanisms underlying the anti-cancer properties of SeNPs have not been fully elucidated, several hypotheses are proposed: (i) increased carcinogen detoxification, oxidative stress, and immune surveillance; (ii) cellular and mitochondria-mediated apoptosis; (iii) inhibited angiogenesis and tumour cell invasion; (iv) S phase cell cycle arrest; (v) inhibited expression of the matrix metalloproteinases preventing metastasis; and (vi) mobilisation of endogenous copper [121,122,123]. Among these possible mechanisms, apoptosis receives the most attention for SeNPs’ anti-cancer activity [124]. SeNPs conjugated with organic molecules and drugs inhibit the accumulation of nanoparticles, increase their anti-cancer efficacy, and alleviate the toxic effects of antibiotics [123,125,126]. SeNPs linked with Spirulina polysaccharides prevent tumour growth through apoptosis confirmed by increased sub G1 cell population, chromatin condensation, and DNA fragmentation. These conjugates also aid SeNPs in the targeted delivery in cancer cells via specific interactions between lectins and carbohydrates present on the cell surface [125].

## 6. Discussion

The search for effective chemopreventative and therapeutic compounds that have minimal or no side effects is currently ongoing. Cancer is an epigenetic disease that arises from the excessive activation of oncogenes and inhibition of tumour suppressor genes. As discussed in this review, organoselenium compounds can modulate gene expression by regulating the epigenome. This can occur by functioning as histone deacetylase inhibitors and modulating the acetylation pattern of histones and non-histone proteins, and it provides the potential for the use of organoselenium compounds as anti-cancer agents. Furthermore, since cancer is an epigenetic disease, the ability of organoselenium compounds to alter the epigenome may increase its efficacy as an anti-cancer agent.

Unlike the current anti-cancer drugs that do not selectively target cancer cells, organoselenium compounds have demonstrated cytotoxic activity against cancer cells whilst leaving non-cancerous cells relatively unharmed. As such, the therapeutic use of organoselenium compounds provides a targeted approach [126]. Previous reports emphasise the use of organoselenium compounds administered in combination with conventional chemotherapeutic treatments [16]. This characteristic of organoselenium compounds may reduce the side effects often associated with the current cancer treatments [127]. However, whether this predisposes non-cancerous cells to develop a carcinogenic phenotype remains to be elucidated.

Furthermore, the excessive intake of organoselenium compounds, above the recommended dietary intake of 400 µg/day for adults, has been associated with toxicity. Other challenges include the bioavailability of the exact concentration of organoselenium compounds required to reverse the epigenetic modification in cancer cells and the effect of organoselenium compounds in combination with existing anti-cancer drugs [128]. The gut microflorae significantly influences the bioavailability of Se; thus, we can manipulate Se nutritional availability [129]. However, the overuse or prolonged use of antibiotics compromises the microbiota and is associated with an excess incidence of cancer diagnosis [130].

These issues can be curbed using SeNPs, which exhibit lower toxicity and have greater bioavailability and biological activity than both natural and synthetic organoselenium compounds [131]. For these reasons, selenium nanoparticles may provide the potential for precision cancer therapy. Several recent studies have highlighted the impact of SeNPs in cancer therapy with optimistic results [132,133,134,135,136,137]. As such, research in this revolutionary field is growing rapidly; however, a better understanding of non-cancerous cells’ interactions must be evaluated.

## 7. Conclusions

Ultimately, through simple dietary choices, such as incorporating foods that are rich in selenium into the diets of cancer patients as well as those patients that are at a high risk of developing cancer, organoselenium compounds may have the potential to decrease the prevalence of cancer and increase patient survival. Likewise, SeNPs can be used as a food additive, and synthetic organoselenium compounds and SeNPs can be used to create nutritional supplements that are easily administered to cancer patients and at-risk individuals with selenium deficiencies.

## Data Availability

Not applicable.
